# Weight gain and incident obesity among male snus users

**DOI:** 10.1186/1471-2458-11-371

**Published:** 2011-05-23

**Authors:** Jenny Hansson, Maria Rosaria Galanti, Cecilia Magnusson, Maria-Pia Hergens

**Affiliations:** 1 Department of Public Health Sciences, Karolinska Institutet, 171 76 Stockholm, Sweden; 2 Department of Medical Epidemiology and Biostatistics, Karolinska Institutet, Stockholm, Sweden

## Abstract

**Background:**

Snus is a moist smokeless tobacco product which has recently reached beyond its original market of Scandinavia. Snus is now being increasingly used in both the United States and South Africa. The effect of snus use on weight is unknown. This study has therefore investigated the relationship between the use of snus, weight gain (≥5%) and the incidence of obesity (body mass index ≥30 kg/m^2^).

**Methods:**

The study participants (n = 9,954 males living in Stockholm County, Sweden) were recruited in 2002 and reassessed in 2007. Tobacco use was categorized according to information obtained in both the baseline and follow-up surveys. Outcomes were assessed by comparing self-reported weight and body mass index between the baseline and follow-up surveys.

**Results:**

Stable current snus use (according to both surveys), compared to never having used any kind of tobacco, seemed to be associated with both weight gain (odds ratio = 1.31, 95% confidence interval: 1.04-1.65) and incident obesity (odds ratio = 1.93, 95% confidence interval: 1.13-3.30) after adjustment for age, baseline weight, alcohol consumption, physical activity, education, consumption of fruit and berries, and the frequency of having breakfast. No associations with incident obesity or weight gain were seen for stable former users of snus (according to both surveys) or among men who quit or began using snus during follow-up.

**Conclusions:**

These data suggest that the use of snus is moderately associated with weight gain and incident obesity among men.

## Background

Snus, a moist smokeless tobacco product used orally, is receiving growing attention in public health research and debate. This is probably due to the increasing use of the product and the ban on smoking in public areas.

Snus has historically been used mainly by men in Sweden, and to some extent in other Scandinavian countries [1]. However, it has now also reached new markets, for example the United States and South Africa [2]. The potential health effects of snus use have not been thoroughly investigated, and the relationship between the use of snus and metabolic processes such as the regulation of body weight is unclear. However, research on the effect of smoked tobacco (with an exposure to nicotine comparable with snus use) [3,4] has found that smokers have a lower body mass index (BMI) than to non-smokers [5-7], possibly as a consequence of increased energy expenditure. Smoking may also affect the body fat distribution and it is associated with abdominal obesity [6,7], impaired glucose tolerance and insulin resistance [5-7]. It is unclear whether the metabolic effects are due to nicotine or to other constituents in tobacco smoke [6]. With regard to snus use, one prospective study reported an association with the onset of obesity [8] while another failed to detect any differences in weight gain between snus users and non-tobacco users [9]. Results of cross-sectional studies of BMI [4,9-15] and abdominal obesity are conflicting [10,15].

To contribute to an understanding of the potential role of snus use on changes in body weight, we studied the association between the use of snus and weight gain (≥ 5%) as well as incidence of obesity (BMI ≥30 kg/m^2^) during a five year follow-up among men in a population-based cohort study set in Stockholm County.

## Methods

### Study population

**Figure 1:**
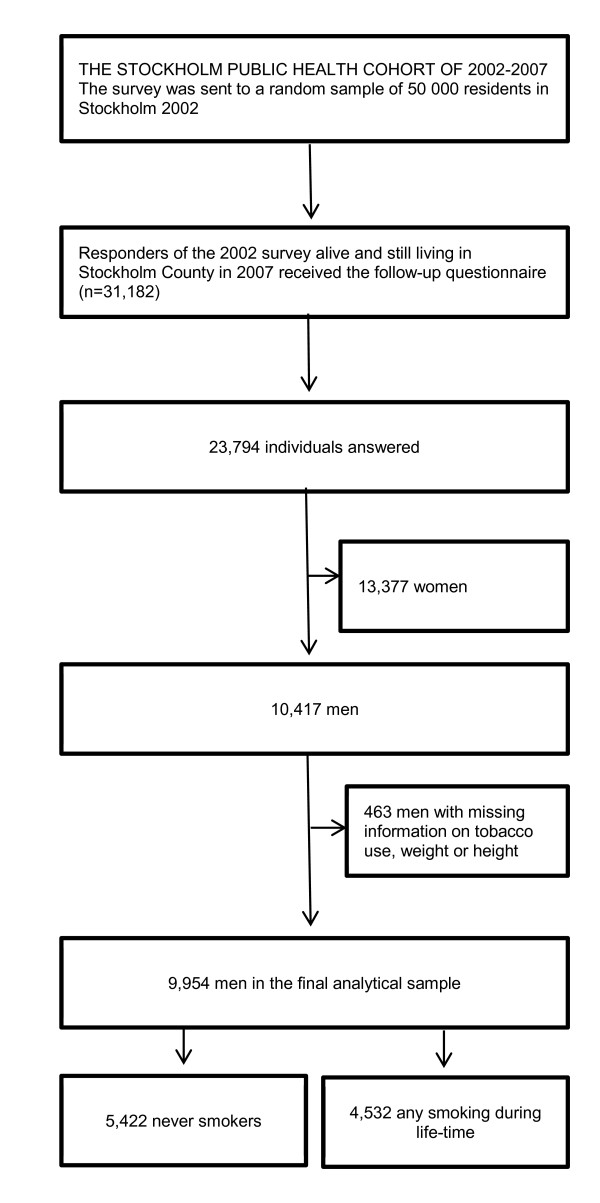
**Derivation of analytical sample**. Flowchart of the study population and the final analytical sample

**Figure 2:**
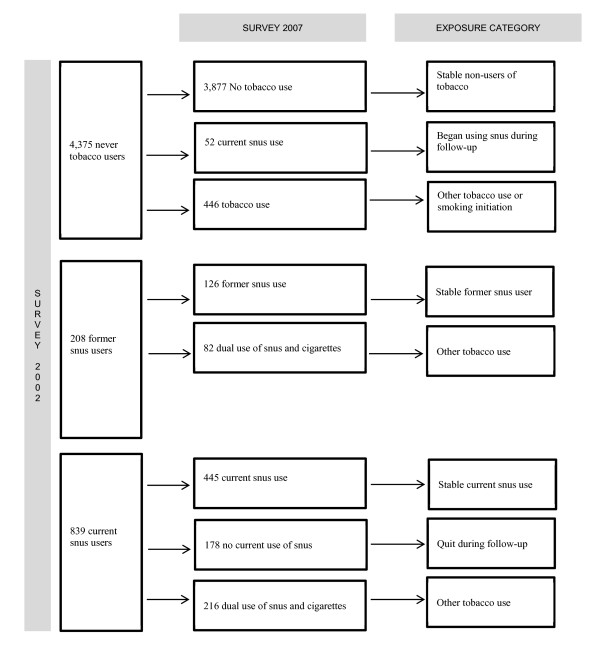
**Information on snus use in 2007 among never-smokers in 2002 (n = 5,422)**. Transitions in tobacco use between baseline and follow-up among never-smokers in 2002, and their corresponding exposure categories employed for analyses of snus use and weight gain/onset of obesity

In 2002, the Stockholm County Council Public Health Survey was sent to a random sample of 50,000 Stockholm County residents, aged 18 to 84 years. Responders (n = 31,182) were asked to participate in a follow-up survey in 2007. Participants in both surveys, 23,794 individuals (corresponding to a 76% retention rate), constitute the Stockholm Public Health Cohort. Due to the low prevalence of snus use among women in this population (1% of women at baseline were snus users who had never smoked) the analyses were restricted to men (n = 10,417). After further exclusion of subjects with missing information regarding tobacco use, weight or height, the final analytical sample contained 9,954 men. Figure 1 describes the derivation of the analytical sample, and Figure 2 details the definition of exposure categories for snus use seen in Table 1. The study was approved by the Regional Ethics Committee in Stockholm.

**Table 1:** Baseline Characteristics According to Tobacco use at Baseline and at Follow-up

	*Never tobacco*	*Daily snus use*				*Daily smoking*				*Other*	*All*
	
*Tobacco use at baseline and follow-up*	No tobacco use	Stable current use	Stable former use	Quit during follow-up	Began during follow-up	Stable current use	Stable former use	Quit during follow-up	Began during follow-up	Including combined snus use and smoking	
n (%)	3877 (39.0)	445 (4.5)	126 (1.3)	178 (1.8)	52 (0.5)	729 (7.3)	1541 (15.5)	284 (2.9)	56 (0.6)	2666 (26.8)	9954
											
Mean age (years)	46.3	36.6	44.3	36.0	31.7	52.6	60.6	50.5	53.7	48.7	49.1
											
Mean weight (kilograms)	81.3	82.2	84.1	83.6	81.8	80.6	83.7	81.6	82.5	83.8	82.4
											
Mean weight gain (kilograms) (SD)	0.7 (4.8)	1.9 (5.1)	1.3 (4.2)	1.2 (4.7)	1.5 (4.3)	0.5 (5.5)	-0.1 (4.7)	2.7 (5.9)	-0.6 (5.7)	0.9 (5.6)	0.7 (5.1)
											
Mean BMI (kg/m^2^)	25.1	25.2	25.6	25.3	24.8	25.3	26.2	25.4	25.8	25.9	25.5
											
Alcohol consumption											
Heavy (%)	19.6	42.8	28.6	29.8	26.9	38.0	29.1	30.0	28.6	36.1	28.6
Moderate (%)	74.9	55.1	69.8	67.3	69.2	53.8	67.0	64.6	65.3	59.4	66.5
None (%)	5.5	2.1	1.7	2.9	3.9	8.1	3.9	5.4	6.1	4.5	4.9
											
Breakfast											
Daily (%)	84.6	57.3	79.2	65.0	64.7	60.7	88.6	68.6	79.3	73.2	78.2
Weekly (%)	11.64	30.7	16.8	26.0	31.4	23.4	7.7	23.6	13.2	17.8	15.2
Never (%)	3.8	11.9	4.0	9.0	3.9	15.9	3.6	7.9	7.6	9.0	6.6
											
Education											
University (%)	45.4	34.8	46.0	45.8	40.4	23.6	33.9	29.9	37.7	30.5	37.1
Upper secondary school (%)	43.4	55.9	38.9	48.6	55.8	49.4	45.7	48.8	37.7	48.3	46.3
Compulsory school (%)	11.2	9.3	15.1	5.7	3.9	27.0	20.4	21.4	24.5	21.2	16.6
											
Fruit and berries											
Daily (%)	54.3	31.5	60.8	40.7	54.0	33.5	59.0	40.7	41.1	43.1	48.9
Occasionally (%)	44.3	63.5	38.4	57.6	46.0	59.9	39.4	53.6	57.1	53.2	48.4
Never (%)	1.3	5.1	0.8	1.7	-	6.7	1.6	5.7	1.8	3.7	2.7
											
Physical activity											
Regular exercise (%)	18.8	19.4	23.4	25.4	40.8	5.6	12.4	7.6	13.0	13.2	15.3
Moderate exercise (%)	70.5	64.0	73.4	70.6	51.0	64.1	75.7	69.3	77.8	70.0	70.4
No exercise (%)	10.7	16.7	3.2	4.0	8.2	30.3	11.9	23.1	9.3	16.8	14.3
											

### Data collection

Baseline data were collected by postal questionnaires, while participants were offered a choice of a postal or a web-based questionnaire at follow-up. In the baseline survey, regular snus use was assessed through the questions "Do you use snus daily?" and "Have you previously used snus daily for at least six months?" Former users also reported the time since cessation. In the follow-up questionnaire, the questions "Have you ever used snus more or less daily for at least a year?" and "Are you currently using snus more or less daily?" were asked. Corresponding questions regarding smoking were included in both surveys. Cessation less than six months prior to baseline was regarded as current use. Subjects were categorized according to tobacco use at baseline and at follow-up, resulting in ten mutually exclusive groups. Never users were subjects who consistently reported that they had never used tobacco daily in both the baseline and follow-up surveys. Exclusive snus users (i.e. snus users who reported that they never had been a regular smoker in both the baseline and the follow-up surveys) were divided into four categories. Stable current users reported use at both baseline and follow-up, while stable former users had quit more than six months prior to the baseline survey and reported that they were still former users at follow-up. Two additional categories included those who started using snus during follow-up (starters) and those who stopped using snus during follow-up (quitters). Exclusive smokers were grouped according to the same principles. The tenth category included all other combinations, including mixed tobacco use, i.e. subjects who during their life-time had used snus and had also smoked regularly.

In both surveys, the respondents reported their height in centimetres and weight in kilograms. Baseline information on leisure time physical activity was divided into: no exercise, moderate exercise and regular exercise. Alcohol consumption (reported as average consumption during weekdays and at weekends) was recalculated into grams of alcohol consumed daily and categorized as never, moderate and heavy consumption (0, 1-24, >24 grams/day). Achieved education was divided into three categories: compulsory school, upper secondary school and university. Frequency of eating breakfast was categorized as never, weekly (one to five times a week) and daily. Consumption of fruit and berries was categorized as never, occasionally (from 1-3 times a month to a couple of times a week) and daily.

### Statistical analyses

Outcome was defined as a weight gain of ≥5% during follow-up, and was assessed by comparing the weight in 2002 with the weight in 2007. In addition, we analysed the odds of becoming obese (BMI ≥30 kg/m^2^) during follow-up among non-obese subjects at baseline (n = 9,028). The associations between snus use and weight gain or incident obesity were explored using logistic regression models and presented as odds ratios (OR) with 95% confidence intervals (CI).

Age and baseline weight were included in the analyses as continuous variables and leisure time physical activity, alcohol consumption, educational level, frequency of eating breakfast, and consumption of fruit and berries in categories as described above. All the analyses were run using SAS version 9.1 (SAS Institute Inc., Cary, N.C., USA).

## Results

Baseline characteristics of the study participants are described in Table 1, and Figure 2 shows how the exposure categories concerning snus users were derived. In total, 18% of participants (n = 1,793) reported current snus use at baseline but among these only 839 were never-smokers (i.e. exclusive snus users). At follow-up, 445 were still exclusive snus users, 178 had quit using snus and 216 reported mixed tobacco use (i.e. both snus use and smoking). Such mixed use was categorised as "Other". This group also included mixed users at baseline and smokers in 2002 that began using snus during follow-up. Out of the 44% (n = 4,375) who had never used tobacco regularly at baseline, 3,877 were still never users of tobacco at follow-up. Fifty-two men had started using snus (categorised as "Began during follow-up").

In the group of stable snus users (n = 445, corresponding to 4.5%) the mean weight was 82.2 kilograms and the mean BMI was 25.2, which were slightly higher than the values for the never users of tobacco. On average, snus users were younger than both never users of tobacco and smokers. Regular physical activity during leisure time was more common in all categories of snus users compared to never users of tobacco and all smokers. Smokers in particular, but also stable current users of snus, less often reported a university degree, than never users of tobacco. An intermediate level of education was most common among the stable current users of snus. The proportion of heavy consumers of alcohol was higher among stable current users of snus than in any other group. Stable current users of snus and stable current smokers had less regular breakfast habits and were less often daily consumers of fruit and berries compared to all the other categories.

During follow-up, stable current snus users gained on average 1.9 kilograms, while never tobacco users gained 0.7 kilograms. The ORs for weight gain according to tobacco use are presented in Table 2. Stable current snus users had a moderately increased OR for weight gain, compared to never tobacco users, after adjustment for age, baseline weight, alcohol consumption, physical activity, education, consumption of fruit and berries, and frequency of having breakfast (OR = 1.31, 95% CI: 1.04-1.65). No significant associations were observed among stable former users of snus, or among men who quit or began using snus during follow-up. Smoking cessation was strongly associated with weight gain (OR = 3.15, 95% CI: 2.39-4.15) and a moderate association was seen among stable current users (OR = 1.24, 95% CI: 1.00-1.54). No associations were seen among stable former smokers or among those who began to smoke during follow-up.

**Table 2:** Odds Ratios and 95% Confidence Intervals for Increase in Body Weight ≥5% in Relation to Tobacco use

Tobacco use 2002 and 2007		Weight gain ≥5% (n/N)	OR (95% CI) ^1^	OR (95% CI) ^2^	OR (95% CI) ^3^
Never tobacco	No tobacco use	790/3877	Referent	Referent	Referent
					
Daily snus use	Stable current use	139/445	1.39 (1.12-1.73)	1.41 (1.13-1.75)	1.31 (1.04-1.65)
					
	Stable former use	31/126	1.24 (0.82-1.89)	1.29 (0.85-1.95)	1.36 (0.89-2.10)
					
	Quit during follow-up	51/178	1.21 (0.86-1.69)	1.24 (0.88-1.74)	1.25 (0.88-1.77)
					
	Began during follow-up	14/52	0.98 (0.52-1.82)	0.98 (0.53-1.84)	0.97 (0.50-1.86)
					
Daily smoking	Stable current use	174/729	1.52 (1.25-1.84)	1.50 (1.24-1.82)	1.24 (1.00-1.54)
					
	Stable former use	235/1541	1.10 (0.93-1.31)	1.13 (0.95-1.34)	1.04 (0.87-1.25)
					
	Quit during follow-up	120/284	3.43 (2.65-4.42)	3.44 (2.66-4.44)	3.15 (2.39-4.15)
					
	Began during follow-up	9/56	0.93 (0.45-1.93)	0.93 (0.45-1.94)	0.70 (0.29-1.67)
					
Ohter	Including combined snus use and smoking	676/2666	1.46 (1.29-1.64)	1.50 (1.33-1.69)	1.34 (1.17-1.53)

^1 ^Adjusted for age.

^2 ^Adjusted for age and baseline weight.

^3 ^Adjusted for age, baseline weight, alcohol consumption, physical activity, education, consumption of fruit and berries, and frequency of having breakfast.

Stable current snus use was associated with incident obesity (OR = 1.93, 95% CI: 1.13-3.30) (Table 3). Neither stable former use nor cessation during follow-up appeared to be associated with the development of obesity. No cases of obesity were observed among men who began to use snus during follow-up. Neither stable current nor stable former smoking was associated with incident obesity. However, quitting smoking during follow-up seemed to be associated with incident obesity (OR = 1.87, 95% CI: 0.99-3.53) as was the uptake of smoking (based on 6 cases).

**Table 3:** Odds Ratios and 95% Confidence Intervals for Developing Obesity in Relation to Tobacco use

Tobacco use 2002 and 2007		Obese cases (n/N)	**OR (95% CI) **^**1**^	**OR (95% CI) **^**2**^	**OR (95% CI) **^**3**^
Never tobacco	No tobacco use	105/3607	Referent	Referent	Referent
					
Daily snus use	Stable current use	21/410	1.68 (1.03-2.72)	1.77 (1.06-2.95)	1.93 (1.13-3.30)
					
	Stable former use	3/112	0.91 (0.28-2.90)	0.74 (0.22-2.46)	0.85 (0.25-2.88)
					
	Quit during follow-up	8/167	1.56 (0.74-3.27)	1.04 (0.48-2.26)	1.13 (0.51-2.50)
					
	Began during follow-up	0/47			
					
Daily smoking	Stable current use	26/653	1.46 (0.94-2.26)	1.71 (1.07-2.72)	1.31 (0.78-2.22)
					
	Stable former use	47/1352	1.35 (0.93-1.95)	1.18 (0.80-1.72)	1.09 (0.72-1.66)
					
	Quit during follow-up	16/260	2.26 (1.32-3.90)	2.37 (1.33-4.23)	1.87 (0.99-3.53)
					
	Began during follow-up	6/53	4.49 (1.87-10.76)	3.94 (1.52-10.21)	3.53 (1.24-10.09)
					
Other	Including combined snus use and smoking	107/2367	1.61 (1.22-2.12)	1.44 (1.08-1.92)	1.25 (0.91-1.73)

^1 ^Adjusted for age.

^2 ^Adjusted for age and baseline weight.

^3 ^Adjusted for age, baseline weight, alcohol consumption, physical activity, education, consumption of fruit and berries, and frequency of having breakfast.

## Discussion

In this study, based on a five-year follow-up of the Stockholm Public Health Cohort, the current use of snus was found to be moderately associated with weight gain and incident obesity. Previously, three longitudinal studies explored the association between snus use and weight. Two of these studies, both based on the Västerbotten Intervention Programme, reported results similar to ours. One observed that the heavy use of snus (>4 cans/week) was associated with incident obesity (OR = 1.70, 95% CI: 1.36-2.18), when the outcome was assessed 10 years after baseline [8]. In the second study, non-users of snus were reported to have a greater chance of maintaining their weight than snus users [16]. The third study found no differences in weight gain between exclusive snus users and non-users of tobacco [9]. However, in a cross-sectional analysis of the same study population, the prevalence ratio for BMI ≥27 among exclusive snus users was slightly elevated as compared to non-users of tobacco. Similarly, another cross-sectional study found that snus users had a higher prevalence of overweight (defined as BMI >26) than non-users of tobacco [12]. Further, one study found a higher mean BMI among snus users than among non-users [17], while four other studies found no differences in mean BMI [4,10,11,14], of which two were partly based on the same study population [4,11]. The use of snus has also been associated with obesity in a cross-sectional analysis of controls in a case-control study [13]. One study investigated the relationship between snus use and obesity based on three different measures (BMI ≥30, waist-hip-ratio ≥1.0 and waist circumference >102 centimetres) [15]. The researchers found no association between exclusive snus use and obesity based on any of these measures. However, a positive association between snus use and waist-hip-ratio has been reported previously [10].

The BMI at baseline did not differ between stable smokers and never users of tobacco, but the findings indicate that weight gain among smokers was higher than among never users of tobacco. This association persisted (although it was weakened) after adjusting for several life-style factors.

Discrepancies in results between different studies may be due to differences in sample size, differences in definition of exposure and/or outcome, or to differences in adjustment of potential confounders, especially cigarette smoking. Five studies presented results for exclusive snus users, (i.e. never smokers) [9,11,12,14,15], five included current and/or former smokers [4,8,10,13,17], whereof three presented results controlled for smoking [8,13,17]. One longitudinal study did not clearly report exclusion or adjustment for smoking [16]. Previous studies with large sample size (>10,000) and large numbers of exposed individuals (>700) all found a positive association between snus use and the respective outcome measure. These studies include the two longitudinal studies based on the Västerbotten Intervention Programme [8,16], and two cross-sectional studies based on the Construction Workers Cohort [12] and the Malmö Diet and Cancer Study [17]. In studies of exclusive snus users, results were conflicting [9,11,12,14,15].

Little is known about the metabolic effects of snus use. However, cigarette smoking is associated with abdominal obesity [6,7], increased cortisol secretion [18-20] and a risk for type 2 diabetes [21]. If these metabolic effects can be attributed to the effect of nicotine on the central nervous system [20], it could be anticipated that they would be similar or more pronounced among snus users, due to similar levels of and sustained exposure to nicotine from this type of tobacco [3,4]. Our finding, of very similar ORs for weight gain among stable current snus users and among stable current smokers, agrees with this hypothesis. Other similarities and differences between metabolic events among smokers and snus users are not easily interpreted. Associations with abdominal obesity [6,7] and impaired glucose tolerance [5-7,21] have been documented for smoking. Among snus users, no increase in waist circumference [10,15] but an increase in waist-hip-ratio [10] has been observed, and results regarding insulin resistance are ambiguous [4,8,11,22,23].

Obesity, overweight and changes in weight can be defined in several ways. We chose to define outcomes as weight gain ≥5% and as incidence of obesity. A weight gain of ≥5% is a suggested cut-off for clinically relevant gain [24]. Obesity, defined by the World Health Organisations as BMI ≥30 [7], is a component of the metabolic syndrome [25] and is often used in studies of weight and health outcomes. It is therefore both clinically relevant and enables comparison with other studies. BMI as a measure of body composition has limitations and does not consider the relative proportions of body fat or muscle mass. Nevertheless, among most individuals a BMI as high as 30 or above is unlikely to be due to muscle mass. The study is based on self-reported information. This is a potential source of bias, which however is likely to be non-differential with regard to tobacco exposure. The low participation rate at baseline, in line with other Swedish studies from the same years, could affect external validity. However, we have no reason to believe that the relationship between snus use and weight seen in this population would be different among men in the general population. Also, there is no reason to believe that attrition at follow-up was differential with regard to both exposure and outcome. The association between smoking cessation and weight gain, in line with previous studies [5,26], suggests no major bias in our results. Strengths of this study include its prospective design and information on several covariates, making it possible to control for potential confounding. We had no information on energy intake, and cannot exclude the possibility of residual confounding. We attempted, however, to control for potential differences in eating habits using available information on the consumption of fruit and berries as well as information on the frequency of eating breakfast, both of which have been associated with energy balance and weight stability [27]. To our knowledge, this is the first study that has attempted to adjust for covariates related to energy intake.

## Conclusion

Our findings suggest that snus use is moderately associated with weight gain and incident obesity. This indicates that there may be an increased risk for metabolic disturbances among snus users, a field that deserves further investigations.

## Competing interests

The authors declare that they have no competing interests.

## Authors' contributions

JH preformed the data analysis and prepared the manuscript. MRG, CM and MPH participated in discussing the analytical strategy and the interpretation of findings, as well as critically revising the manuscript. All authors read and approved the final manuscript.

## Pre-publication history

The pre-publication history for this paper can be accessed here:

http://www.biomedcentral.com/1471-2458/11/371/prepub
